# Trends in Mortality from Ischemic Heart Disease in the Region of the Americas, 2000–2019

**DOI:** 10.5334/gh.1144

**Published:** 2022-08-11

**Authors:** Fernando Lanas, Alvaro Soto

**Affiliations:** 1 Departamento de Medicina Interna, Facultad de Medicina, Universidad de La Frontera, Temuco, Chile; 2 Departamento de Especialidades Médicas, Facultad de Medicina, Universidad de La Frontera, Temuco, Chile

**Keywords:** Americas region, Ischemic heart disease, mortality, Joinpoint

## Abstract

**Background:**

In latest decades, mortality rates from ischemic heart disease (IHD) had declined steadily in most of the world as a consequence of improvements in prevention and therapy.

**Objective:**

The aim of this study was to analyze trends in mortality caused by IHD in the region of the Americas from 2000 to 2019.

**Methods:**

Estimates of the age-adjusted mortality rate (AAMR) due to IHD were extracted from the Data Portal on Noncommunicable Diseases, Mental Health, and External Causes (ENLACE), Pan American Health Organization. We used Joinpoint regression to analyze significant changes in mortality trends by country, gender, geographical sub-region, and country income, according to the World Bank classification. We also calculated the average annual percent change (AAPC) mortality rate for the overall period in the Americas as a whole and by country and sub-region.

**Results:**

In the region of the Americas, the AAMR from IHD decreased from 117.80 (95% uncertainty interval (UI)) 106.64–135.90) in 2000 to 73.64 (62.65–92.66) per 100,000 in 2019. In males, from 149.08 (95% UI 138.23–168.08) to 96.02 (95% UI 83.48–117.19) and in females 92.36 (95% UI 81.35–109.42) to 54.84 (95% UI 45.28–71.76). The AAPC mortality rate in the region decreased –2.5% (95% CI: –2.7, –2.3), with joinpoints in 2007 and 2012, –2.3% (95% CI: –2.5, –2.1) in men and –2.7% (95% CI: –3.0, –2.5) in women. According to the sub-region analysis, the highest decrease was recorded in North America, AAPC –3.1% (95% CI: –3.3, –3.0) with one joinpoint in 2011, whereas there was a stagnation of the mortality rate in Central America, Mexico, and Latin Caribbean with an AAPC of 0.1 (–0.2, 0.3) with one joinpoint in 2007.

**Conclusions:**

Age-adjusted mortality rate from IHD between 2000 and 2019 has decreased in the region of the Americas. However, different trends were observed, North America had the highest reduction in AAPC, while Central America, Mexico, and Latin Caribbean Region had a stagnation. This trend was highly influenced by country income.

## Introduction

Cardiovascular diseases are the leading cause of death in the region of the Americas and in the world. Ischemic heart disease (IHD) has become the first isolated cause of death [1] despite the fact that the risk factors for the majority of IHD events are known, easy to identify and potentially treatable [2]. In Latin America rheumatic heart disease and Chagas disease, once a major health problem in the region of the Americas, are now responsible for only 1% of the mortality [3].

As a consequence of the world’s population growth and the increase in life expectancy, an increase in the total number of deaths from IHD has been observed worldwide [4]. In North American countries the total population increased between 2000 to 2020 from 312.8 to 369.2 million inhabitants and life expectancy changed from 76.9 to 79.2 years. In Latin America and the Caribbean countries, the population increased from 520.9 million to 652.3 million, and the life expectancy from 71.7 to 75.3 years [5]. However, the estimated death by IHD increased only slightly from 1.024,117 (18.8%) in 2000 to 1.091.311 (15.2%) in 2019 [6]. Mortality rates from IHD have declined steadily in most of the world in recent years [4] as a consequence of preventive interventions and improvements in therapy. Although, the increase in risk factors such as obesity, metabolic syndrome, and diabetes mellitus has reduced the potential benefit of these interventions. In the region of the Americas, the tendencies in mortality rates due to IHD have been variable between countries, for example, it has been reported a significant decrease in Argentina, a discrete decrease in Colombia in contrast to an increase in Mexico [7]. The aim of this study was to analyze trends in mortality caused by IHD in the region of the Americas countries from 2000 to 2019.

## Methods

A secondary data analysis was performed using the Data Portal on Noncommunicable Diseases, Mental Health, and External Causes (ENLACE) from the Pan American Health Organization [8]. Age-adjusted mortality rate (AAMR) data for men and women, with its correspondent 95% uncertainty interval (95% UI), from the region of the Americas countries in the period 2000 to 2019 were extracted. The data source was WHO Global Health Estimates [9]. WHO technical documents the data sources and methods used for preparation of the country-level Global Health Estimates [10]. The average world population age structure constructed for the period 2000–2025 was used for standardizing the country mortality rates [11].

In the ENLACE database, sub-regions and countries with available data included only countries that are WHO Member States with a population over 90,000 in 2019: North America (Canada, United States of America), Mexico and Central America (Belize, Costa Rica, El Salvador, Guatemala, Honduras, Nicaragua, Panama), Latin Caribbean (Cuba, Dominican Republic, Haiti), Andean Area (Bolivia, Colombia, Ecuador, Peru, Venezuela), Brazil, Southern Cone (Argentina, Chile, Paraguay, Uruguay) and Non-Latin Caribbean (Antigua and Barbuda, Bahamas, Barbados, Grenada, Guyana, Jamaica, Saint Lucia, Saint Vincent and the Grenadines, Suriname, Trinidad and Tobago. The database did not have mortality rates from the following countries: Anguilla, Bermuda, Curacao, Dominica, French Guiana, Guadeloupe, Martinique, Monserrat, Puerto Rico, Saint Kitts and Nevis, Saint Maarten (Dutch part), Turks and Caicos Islands, Virgin Islands (British) and Virgin Islands (U.S.).

We also conducted an analysis to assess regional mortality trends by sex, sub-region, and income group, according to the World Bank income classification for the fiscal year 2012 (data for the calendar year 2010) [11]. American countries were classified into four income categories: high-income (Bahamas, Barbados, Canada, Trinidad and Tobago, and United States), upper-middle-income (Antigua and Barbuda, Argentina, Brazil, Chile, Colombia, Costa Rica, Cuba, Dominican Republic, Ecuador, Grenada, Jamaica, Mexico, Panama, Peru, Saint Lucia, St Vincent, Suriname, Uruguay, and Venezuela), lower-middle-income (Belize, Bolivia, El Salvador, Guatemala, Guyana, Honduras, Nicaragua, and Paraguay), and low-income (Haiti). The median AAMR with its correspondent interquartile range (IQR) were calculated by year and income category.

We used the Joinpoint regression software (version 4.8.0.1; Surveillance Research Program, USA National Cancer Institute, Bethesda, MD, USA) to analyze significant changes in mortality trends. This analysis identified inflection points (called ‘joinpoints’) at which there was a significant change in the linear slope of the trend. The Joinpoint regression analysis has been used extensively in previous research on trends in cardiovascular and cerebrovascular disease [12,13,14]. The number and the location of significant joinpoints for each country were determined using a log-linear model. We computed the estimated annual percent change (APC), and corresponding 95% confidence intervals (CI), to describe the magnitude of change for each of the trends identified. In this model, age-standardized mortality rates were used as the dependent variable and year of death as the independent variable with an annual interval type and assuming a constant variance (homoscedasticity). In all analyses, P < 0.05 was regarded as statistically significant. We also calculated the average annual percent change (AAPC) for the overall period (2000–2019) in the region of the Americas as a whole, by sub-region, and by country. Due to the study design, no approval by an institutional review board was required.

## Results

The overall AAMR decreased from 117.80 (95% UI: 106.64–135.90 in 2000 to 73.64 (95% UI: 62.65–92.66) per 100,000 in 2019. In 2000, the highest rate was found in Guyana with 215.18 (95% UI: 180.32–252.79) per 100,000 and the lowest in Jamaica with 52.32 (95% UI: 44.47–59.39) per 100,000. In 2019 Guyana, had the highest mortality rate with 192.88 (95% UI: 147.90–259.09) per 100,000, whereas the lowest rate was observed in Chile with 36.69 (95% UI: 31.66–49.81) per 100,000) (Table 1S).

**Figure 1:**
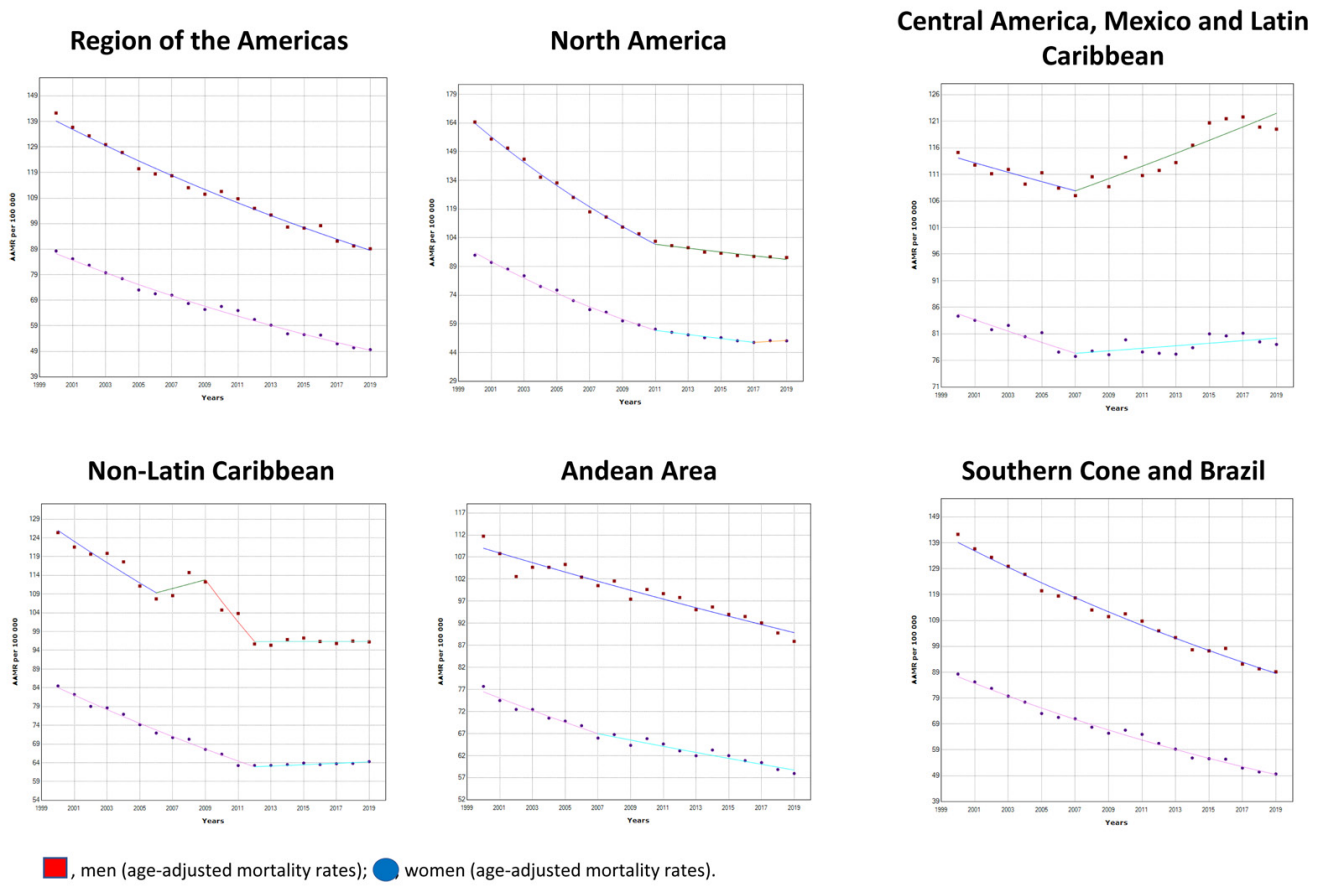
Trends in mortality from ischemic heart diseases in the Region of the Americas, 2000–2019.

During the period 2000–2019, the region of the Americas showed a statistically significant decrease of –2.5% (95% CI: –2.7, –2.3) in the AAPC in mortality rates, with two joinpoints in 2007 and 2012 due to a lower AAPC after these years (Table 1, Figure 1). Mortality rates decreased significantly in twenty-four countries. Trinidad Tobago, Costa Rica, and Canada had the largest decreases (AAPC –4.3%, –4%, and –3.9%, respectively). Mortality rates increased significantly in three countries. Dominican Republic, Grenada had Mexico (AAPC 2.4%, 1.4%, and 0.6%, respectively) (Table 2).

**Table 1:** Joinpoint analysis for ischemic heart diseases mortality trends in the Region of the Americas, sub-regions and income categories, 2000–2019.


SUB REGION	TOTAL STUDY PERIOD	PERIOD 1		PERIOD 2		PERIOD 3	
			
	AAPC (95% CI)	YEARS	APC (95% CI)	YEARS	APC (95% CI)	YEARS	APC (95% CI)

Region of the Americas	–2.5* (–2.7, –2.3)	2000–2007	–3.7* (–4.0, –3.4)	2007–2012	–2.6* (–3.2, –2.0)	2012–2019	–1.1* (–1.4, –0.8)

North America	–3.1* (–3.3, –3.0)	2000–2011	–4.5* (–4.7, –4.3)	2011–2019	–1.2* (–1.5, –0.8)		

Central America, Mexico and Latin Caribbean	0.1 (–0.2, 0.3)	2000–2007	–1.0* (–1.7, –0.4)	2007–2019	0.7* (0.4, 1.0)		

Andean Area	–1.2* (–1.3, –1.0)						

Southern Cone and Brazil	–2.6* (–2.7, –2.5)						

Non-Latin Caribbean*	–1.3* (–2.1, –0.6)	2000–2009	–1.8* (–2.2, –1.3)	2009–2012	–3.2 (–7.9, 1.8)	2012–2019	0.1 (–0.6, 0.7)

**Income category**							

High income	–2.1* (–2.5, –1.6)	2000–2014	–2.6* (–2.9, –2.3)	2014–2019	–0.5 (–2.0, 1.0)		

Upper-middle income	–1.3* (–1.5, –1.1)						

Lower-middle income	–0.9* (–1.5, –0.4)	2000–2006	–2.9* (–4.4, –1.3)	2006–2019	–0.1 (–0.5, 0.4)		

Low income	–0.3* (–0.4, –0.2)						


AAPC, average annual percent change; APC, annual percent change; CI, confidence interval. * P < 0.05 for change in trend.

**Table 2:** Joinpoint analysis for ischemic heart diseases mortality trends in the countries of the region of the Americas from 2000 to 2019.


COUNTRY	TOTAL STUDY PERIOD	PERIOD 1		PERIOD 2		PERIOD 3	
			
	AAPC (95% CI)	YEARS	APC (95% CI)	YEARS	APC (95% CI)	YEARS	APC (95% CI)

Antigua and Barbuda	–0.9 (–1.8, 0.0)	2000–2007	4.0* (1.8, 6.2)	2007–2019	–3.6* (–4.5, –2.7)		

Argentina	–2.6* (–2.9, –2.3)						

Bahamas	–0.8* (–1.4, –0.2)	2000–2014	–1.5* (–2.0, –1.1)	2014–2019	1.2 (–0.9, 3.3)		

Barbados	–2.0* (–3.2, –0.8)	2000–2008	–1.9* (–2.7, –1.0)	2008–2011	–8.5* (–15.7, –0.7)	2011–2019	0.4 (–0.5, 1.3)

Belize	–3.6* (–4.6, –2.5)	2000–2002	–12.1* (–20.3, –3.2)	2002–2012	–4.2* (–5.0, –3.3)	2012–2019	–0.1 (–1.4, 1.2)

Bolivia	–0.5* (–0.7, –0.2)	2000–2002	–3.2* (–5.2, –1.2)	2002–2006	–1.7* (–2.7, –0.6)	2006–2019	0.4* (0.2, 0.5)

Brazil	–2.6* (–2.9, –2.4)	2000–2006	–3.2* (–3.8, –2.5)	2006–2019	–2.4* (–2.6, –2.2)		

Canada	–3.9* (–4.1, –3.8)	2000–2012	–5.0* (–5.2, –4.8)	2012–2019	–2.1* (–2.5, –1.8)		

Chile	–3.4* (–3.6, –3.2)						

Colombia	–1.2* (–2.2, –0.0)	2000–2006	–0.9 (–2.1, 0.4)	2006–2019	–3.4 (–10.1, 3.8)	2009–2019	–0.7* (–1.2, –0.1)

Costa Rica	–4.0* (–5.1, –2.9)	2000–2008	–5.3* (–6.6, –4.0)	2008–2014	0.1 (–2.7, 3.0)	2014–2019	–6.8* (–9.4, –4.1)

Cuba	–1.6* (–2.2, –1.0)	2000–2013	–2.4* (–3.0, –1.9)	2013–2019	0.2 (–1.5, 2.0)		

Dominican Republic	2.4* (1.6, 3.1)	2000–2016	3.6* (3.3, 4.0)	2016–2019	–4.2 (–8.4, 0.3)		

Ecuador	–0.8* (–1.3, –0.3)	2000–2005	1.7* (0.4, 3.1)	2005–2014	–2.3* (–2.9, –1.6)	2014–2019	–0.7 (–1.9, 0.6)

El Salvador	–1.5* (–2.2, –0.7)						

Grenada	1.4* (0.1, 2.6)	2000–2005	6.5* (1.9, 11.2)	2005–2019	–0.4 (–1.3, 0.5)		

Guatemala	–0.9* (–1.6, –0.2)	2000–2002	–4.5 (–10.0, 1.3)	2002–2015	0.1 (–0.3, 0.5)	2015–2019	–2.5* (–4.3, –0.6)

Guyana	–0.5* (–0.7, –0.2)	2000–2004	2.1* (1.2, 3.0)	2004–2014	–1.7* (–2.0, –1.5)	2014–2019	0.1 (–0.6, 0.7)

Haiti	–0.3* (–0.4, –0.2)						

Honduras	0.5 (–0.1, 1.2)	2000–2010	–0.1 (–0.4, 0.3)	2000–2013	8.9* (4.4, 13.5)	2013–2019	–2.5* (–3.2, –1.8)

Jamaica	–0.5 (–3.1, 2.2)	2000–2005	–5.4* (–8.9, –1.7)	2005–2008	5.5 (–10.9, 25.0)	2008–2019	0.2 (–0.9, 1.4)

Mexico	0.6* (0.2, 1.0)	2000–2007	–1.0* (–1.9, –0.1)	2007–2019	1.5* (1.1, 1.9)		

Nicaragua	–0.6 (–2.4, 1.3)	2000–2011	–0.8* (–1.6, –0.0)	2011–2014	3.3 (–8.4, 16.5)	2014–2019	–2.3 (–4.9, 0.3)

Panama	–1.5* (–2.2, –0.8)	2000–2010	0.3 (–0.6, 1.3)	2010–2019	–3.5* (–4.6, –2.4)		

Paraguay	–0.5 (–2.0, 0.9)	2000–2002	–5.9 (–17.1, 7.0)	2002–2009	2.8* (0.6, 5.0)	2009–2019	–1.8* (–2.7, –0.8)

Peru	–1.8* (–2.9, –0.7)	2000–2007	–3.9* (–4.8, –3.0)	2007–2010	4.0 (–3.0, 11.6)	2010–2019	–2.0* (–2.6, –1.4)

Saint Lucia	–1.4* (–1.8, –1.0)	2000–2005	–2.0* (–3.0, –1.0)	2005–2011	–5.1* (–6.0, –4.1)	2011–2019	1.8* (1.3, 2.3)

Saint Vincent	–2.1* (–3.5, –0.7)	2000–2004	–3.9* (–7.5, –0.1)	2004–2008	8.7* (2.3, 15.5)	2008–2019	–5.2* (–5.9, –4.4)

Suriname	–0.2 (–0.8, 0.4)	2000–2011	–2.3* (–2.7, –2.0)	2011–2015	0.8 (–1.7, 3.3)	2015–2019	5.0* (3.4, 6.7)

Trinidad and Tobago	–4.3* (–4.5, –4.0)						

United States of America	–3.0* (–3.2, –2.9)	2000–2011	–4.5* (–4.7, –4.2)	2011–2019	–1.1* (–1.4, –0.7)		

Uruguay	–2.5* (–2.8, –2.3)						

Venezuela	–1.2* (–1.9, –0.5)	2000–2016	–0.8* (–1.1, –0.5)	2016–2019	–3.4 (–7.4, 0.8)		


AAPC, average annual percent change; APC, annual percent change; CI, confidence interval. * P < 0.05 for change in trend.

## Trends of mortality by gender

The overall AAMR decreased from 149.08 (95% UI 138.23–168.08) to 96.02 (95% UI 83.48–117.19) per 100,000 in men. In 2000 the highest rates were observed in Guyana with 250.84 (95% UI 210.23–296.28) per 100,000, and the lowest in Peru with 60.28 (95% UI 47.72–74.14) per 100,000. In 2019, Guyana had the highest rates among men with 228.89 (95% UI 175.84–312.72) per 100,000, whereas the lowest was found in Peru with 46.57 (95% UI 30.41–66.57) per 100,000 (Table 1S, Figure 1)). Throughout the sub-regions of the Americas, men showed a statistically significant decrease in mortality with an AAPC of –2.3% (95% CI: –2.5, –2.1), with two joinpoints in 2007 and 2012 (Table 3). Mortality rates between 2000 and 2020 decreased significantly in twenty countries. Canada, Trinidad and Tobago, and Costa Rica had the largest decreases, whereas we observed a significant increase in two countries: the Dominican Republic and Mexico (Table 3 and 2S).

**Table 3:** Joinpoint analysis for ischemic heart diseases mortality trends in men in the sub regions of the Region of the Americas 2000–2019.


SUB REGION	TOTAL STUDY PERIOD	PERIOD 1		PERIOD 2		PERIOD 3	
			
	AAPC (95% CI)	YEARS	APC (95% CI)	YEARS	APC (95% CI)	YEARS	APC (95% CI)

Region of the Americas	–2.3* (–2.5, –2.1)	2000–2007	–3.5* (–3.8, –3.3)	2007–2012	–2.3* (–2.9, –1.7)	2012–2019	–1.0* (–1.2, –0.7)

North America	–3.0* (–3.1, –2.8)	2000–2011	–4.3* (–4.5, –4.2)	2011–2019	–1.0* (–1.3, –0.7)		

Central America, Mexico and Latin Caribbean	0.4* (0.1, 0.7)	2000–2007	–0.8* (–1.5, –0.1)	2007–2019	1.1* (0.7, 1.4)		

Andean Area	–1.0* (–1.1, –0.9)						

Southern Cone and Brazil	–2.4* (–2.5, –2.2)						

Non-Latin Caribbean*	–1.3* (–2.4, –0.2)	2000–2009	–1.3* (–2.0, –0.7)	2009–2012	–3.9 (–10.6, 3.3)	2012–2019	–0.1 (–1.1, 0.9)


AAPC, average annual percent change; APC, annual percent change; CI, confidence interval. * P < 0.05 for change in trend.

For women, the overall ASDR decreased from 92.36 (95% UI 81.35–109.42) to 54.84 (95% UI 45.28–71.76) per 100,000. The highest rates in 2000 were recorded in Haiti with 196.45 (95% UI 125.88–279.25) per 100,000, and the lowest in Jamaica with 44.93 (95% UI 37.18–51.79) per 100,000. In 2019, we observed the highest female rate in Haiti with 180.35 (95% UI 106.35–273.21) per 100,000 and the lowest in Chile with 24.83 (95% UI 20.60–37.16) per 100,000) (Table 1S, Figure 1). Among women, we observed a steady and statistically significant decrease of –2.7% (95% CI: –3.0, –2.5) in the AAPC in the region of the Americas with two joinpoints in 2009 and 2014. Mortality rates between 2000 and 2020 decreased significantly in twenty-four countries. Costa Rica, Canada, and Chile had the largest decreases, whereas we observed a significant increase in two countries: the Dominican Republic and Grenada (Table 4 and 3S).

**Table 4:** Joinpoint analysis for ischemic heart diseases mortality trends in women in the Region of the Americas (Sub regions), 2000–2019.


SUB REGION	TOTAL STUDY PERIOD	PERIOD 1		PERIOD 2		PERIOD 3	
			
	AAPC (95% CI)	YEARS	APC (95% CI)	YEARS	APC (95% CI)	YEARS	APC (95% CI)

Region of the Americas	–2.7* (–3.0, –2.5)	2000–2009	–3.8* (–4.0, –3.7)	2009–2014	–2.3* (–3.0, –1.6)	2014–2019	–1.1* (–1.6, –0.6)

North America	–3.4* (–3.8, –2.9)	2000–2011	–4.9* (–5.1, –4.6)	2011–2017	–2.0* (–2.8, –1.1)	2017–2019	1.0 (–2.9, 5.0)

Central America, Mexico and Latin Caribbean	–0.3* (–0.5, –0.0)	2000–2007	–1.3* (–1.9, –0.7)	2007–2019	0.3* (0.0, 0.6)		

Andean Area	–1.4* (–1.6, –1.2)	2000–2007	–1.9* (–2.4, –1.4)	2007–2019	–1.1* (–1.3, –0.9)		

Southern Cone and Brazil	–2.9* (–3.1, –2.8)						

Non-Latin Caribbean	–1.4* (–1.5, –1.3)	2000–2012	–2.4* (–2.5, –2.2)	2012–2019	0.3 (–0.0, 0.6)		


AAPC, average annual percent change; APC, annual percent change; CI, confidence interval. * P < 0.05 for change in trend.

## Trends of mortality by sub-region

North America had the largest overall AAMR decrease, from 125.81 (95% UI: 115.83–143.96) to 70.34 (95% UI 63.12–86.07) per 100,000, it also had a greater decrease of –3.1% (95% CI: –3.3, –3.0) in the AAPC, after a joinpoint in 2011. In Southern Cone and Brazil the overall AAMR decreased from 112.53 (95% UI 101.85–132.26) to 67.29 (95% UI 59.29–85.32) per 100,000. The AAPC was –2.6% (95% CI: –2.7, –2.5) without joinpoints. A minor decrease in AAPC was observed in the Non-Latin Caribbean, the Andean Area, and Central America. In the Non-Latin Caribbean sub-region, the overall AAMR decreased from 103.78 (95% UI 90.08–117.15) to 79.49 (95% UI 60.98–102.44) per 100,000, the AAPC was –1.3* (95% CI: –2.1, –0.6) with two joinpoints in 2009 and 2012. In Andean Area the overall AAMR decreased from 93.21 (95% UI 78.86–113.89) to 71.68 (95% UI 51.50–98.75) per 100,000, the AAPC was –1.2* (95% CI: –1.3, –1.0) without joinpoints. Finally, in Central America, Mexico and Latin Caribbean the overall AAMR decreased from 98.67 (95% UI 86.00–117.99) to 97.61 (95% UI: 75.92–128.51) per 100,000, the AAPC was 0.1 (95% CI: –0.2, 0.3) with one joinpoint in 2007 (Table 1, Figure 1).

## Trends of mortality by country income

All income groups, according to the World Bank income classification, had a significant decrease in AAPC. However, the magnitude of the change decreased progressively from high to low-income categories. In high-income American region countries, the median overall AAMR decreased from 106.46 (IQR: 91.71–128.88) to 73.47 (IQR: 55.5–81.57) per 100,000. We recorded a statistically significant decrease of –2.1% (95% CI: –2.5, –1.6) in the AAPC, with a joinpoint in 2014. After 2014 a non-significant decrease in APC was observed. In upper-middle-income countries, the median overall AAMR decreased from 93.39 (IQR: 71.44–113.62) to 75.08 (IQR: 53.53–93.7) per 100,000, and in lower-middle-income countries the median overall AAMR decreased from 116.46 (IQR: 106.24–139.82) to 92.9 (IQR: 79.58–126.79) per 100,000, both with similar AAPC. Finally, low-income countries, represented only by Haiti, had an overall AAMR decrease from 199.63 (95% UI: 132.77–282.24) to 185.42 (95% UI: 114.89–276.57) per 100,000 with the smallest AAPC (Table 1).

## Discussion

In the last 20 -year period, age-adjusted mortality rate from IHD decreased in the region of the Americas, with a large decline in the first half and a modest reduction in the second. However, the mortality tendency differs among the Americas region countries and sub-regions: it decreases in most of the countries and sub-regions and in all income categories. AAPC increased in three countries, and the improvement was limited in the Andean Area, Central America, Mexico and Latin Caribbean, and the Non-Latin Latin Caribbean. Additionally, we observed joinpoints in the adjusted rates in most of the countries. The overall reduction of –2.5% in the AAPC between 2000 and 2019 is consistent with the Global Burden of Diseases initiative estimate of a decrease in the age-standardized death rate for IHD of 11.6% in a ten years period, between 2006 and 2016 [15]. The finding that in the Americas region, age-standardized rates of death from IHD has a different trend between sub-regions mirrors what has been reported in Europe, where in Western European countries, a 30% decline in IHD deaths was registered in a 30-year period, since 1980, but in Eastern Europe, the rate has only minor changes [16]. In the wealthy countries in the region of the Americas, the United States, and Canada a trend similar to Western Europe was observed. In Canada from 1986 to 2000 the AAPC declined by 3.44 percent for males and 3.42 percent for females [17]. In the United States, the age-adjusted mortality rate for IHD declined from 194.6 per 100,000 in 1999 to 109.2 per 100,000 in 2011, but the slope has flattened with a further decline of only to 90.9 per 100,000 in the next seven years [18]. This deceleration in the mortality for IHD rate, observed also in other countries of the Americas Region, reduces the possibility of a major cardiovascular disease burden control [19].

Several explanations for this diverging trend have been proposed: changes in risk factor prevalence, level of education, access to affordable and effective medication, and quality of medical care. Association of risk factor prevalence in a short term and changes in IHD mortality has been documented in Russia after the Communist decline with increased mortality associated with increased levels of smoking, alcoholism, poor diet, and low physical exercise [20]. Similarly, in East Germany, a major IHD mortality, compared with West Germany after unification was associated with higher levels of smoking, cholesterol, hypertension, obesity, and diabetes mellitus [21]. However, a recent analysis of age-standardized rates of death from IHD changes, between 2005 and 2015, in the United Kingdom, United States, Brazil, Kazakhstan, and Ukraine describes a progressive decrease in mortality, with a limited reduction of smoking and hypertension prevalence, but an increase in obesity and diabetes mellitus, suggesting that the driving force was an economic improvement [22]. Significant support for the importance of education and quality of medical as protective factors were raised in an analysis of the Population Urban Rural Epidemiology (PURE) cohort, which includes high, middle, and low-income countries. The incidence of cardiovascular diseases was higher in individuals with low education, although the risk factors prevalence was lower in this group and was independent of the individual wealth estimates. Medical care, including management of hypertension, diabetes, and secondary prevention had an important role in cardiovascular event incidence [23]. Another analysis of the PURE cohort reports that lower availability and affordability of essential CVD medicines were associated with an increased risk of major cardiovascular outcomes [24], other evidence of the importance of medical care quality in cardiovascular prevention.

Gender differences in the prevalence of risk factors, risk factor management in primary and secondary prevention, and cardiovascular risk have been reported. Women have a lower prevalence of smoking and alcohol use, lower waist to hip ratio, and higher HDL cholesterol levels, and they have more obesity and sedentary lifestyle [25,27]. In the general population, women had better control of hypertension but, after a coronary event received less effective medication, and coronary artery interventions [25]. In our results, women had a lower initial and final age-standardized mortality rate, as has been reported in several communications [22,27,28], and the overall reduction AAPC in American countries was also higher in females than in males, –2.7% vs –2.3%, respectively. A similar trend was reported for stroke, with a median AAPC of –2.7% for males and females [29].

This study has several strengths. To our knowledge, this is the most recent study analyzing IHD mortality trends in the whole American Region. On the other hand, mortality data were extracted directly from an official database without calculating mortality from death count and population data. The Joinpoint regression software has been widely used to analyze mortality trends in cardio and cerebrovascular disease [30,31]. In addition, this analysis method achieves a better fit compared to linear models, which reduces the tendency to a single regression [32]. Our work also has limitations. The main limitation is the lack of data from some countries that were not included in the database. Additionally, data quality has been rated insufficient in some countries [15]. Finally, we recognize that mortality trend studies only describe trends and do not aim to explain them [29]. This analysis needs further work to investigate the association between trends in IHD mortality and sociodemographic characteristics, such as measures of income and health care expenditure.

## Conclusion

Age-adjusted mortality rate from IHD between 2000 and 2019 had decreased in the American region. However, different trends were observed, North America, Brazil, and the Southern Cone had the highest reduction in AAPC, the Caribbean Region, Central America, and the Andean Area had a lower reduction, while Mexico had an increase in AAPC. This trend was highly influenced by country income.

## Additional File

The additional file for this article can be found as follows:

10.5334/gh.1144.s1 Supplementary Tables. Tables 1S to 3S.
