# Supplementary material for: Trends in Mortality from Ischemic Heart Disease in the Region of the Americas, 2000–2019
Source: Glob Heart. 2022 Aug 11;17(1):53. doi: 10.5334/gh.1144 (PMC9374038; doi:10.5334/gh.1144)
Supplement: Supplementary Tables. — Tables 1S to 3S. [file gh-17-1-1144-s1.pdf]

Supplementary tables

Table 1 S. Ischemic heart disease age-adjusted mortality rate (per 100.000 population) with 95% uncertainty interval (UI).

Country	Total				Male				Female			
	2000		2019		2000		2019		2000		2019	
	Value	95 % UI	Value	95 % UI	Value	95 % UI	Value	95 % UI	Value	95 % UI	Value	95 % UI
Region of the Americas	117.80	106.64, 135.90	73.64	62.65, 92.66	149.08	138.23, 168.08	96.02	83.48, 117.29	92.36	81.35, 109.42	54.84	45.28, 71.76
Argentina	123.20	109.32, 154.19	75.08	64.87, 94.11	168.09	152.12, 204.05	102.79	91.14, 124.01	88.70	76.72, 115.18	53.83	44.90, 70.81
Antigua and Barbuda	89.94	75.54, 104.72	77.56	61.83, 93.99	106.67	91.07, 122.19	89.47	71.81, 107.77	75.58	62.25, 89.68	67.25	53.18, 82.05
Bahamas	106.46	90.64, 121.32	89.08	69.50, 111.49	140.42	121.06, 158.29	117.90	92.52, 147.11	81.02	67.97, 93.51	65.13	50.45, 81.74
Belize	147.17	129.25, 164.51	73.40	59.80, 87.59	181.12	160.79, 200.99	87.52	71.97, 103.79	114.98	99.35, 129.94	59.14	47.49, 71.24
Bolivia	111.12	69.09, 164.13	100.57	61.24, 148.50	127.40	82.23, 182.06	112.28	70.07, 162.99	96.91	57.75, 148.26	90.06	53.48, 135.22
Brazil	113.62	103.81, 130.89	68.12	60.68, 86.14	139.50	129.69, 156.41	89.59	81.19, 109.49	91.90	82.22, 109.54	50.71	44.07, 67.01
Barbados	83.44	71.63, 94.99	55.50	44.24, 70.90	100.31	86.95, 113.68	65.57	52.64, 79.16	69.05	58.67, 78.97	46.69	36.95, 64.11
Canada	97.71	87.74, 108.52	45.14	38.71, 52.82	134.02	123.52, 147.02	61.45	54.39, 69.56	69.06	59.88, 77.60	30.63	24.92, 37.79
Chile	71.44	63.64, 80.25	36.69	31.66, 49.81	95.56	86.79, 105.68	51.21	45.23, 65.30	53.53	46.53, 61.30	24.83	20.60, 37.16
Colombia	100.20	88.73, 127.05	76.07	56.58, 108.45	118.11	105.81, 143.19	93.47	69.96, 131.47	85.75	74.92, 114.57	62.14	45.96, , 9.86

Costa Rica	93.39	81.92, 103.03	40.73	30.54, 55.13	124.48	110.61, , 35.74	55.11	41.63, 76.56	67.87	58.22, 76.22	27.78	20.62, 35.33
Cuba	131.70	119.56, 143.98	97.61	78.30,125.20	156.36	143.36, 172.20	118.13	94.92, 156.52	109.03	97.71, 117.71	79.04	63.29, 95.93
Dominican Republic	97.93	78.74, 118.20	144.37	103.77, 192.18	124.07	100.52, 150.28	174.66	124.88, 234.34	72.45	57.60, 86.91	116.38	84.28,153.19
Ecuador	70.44	59.53, 80.80	57.97	43.78, 75.40	85.47	73.27, 97.26	73.11	55.04, 96.05	57.01	47.27, 66.06	44.72	33.98, 57.31
Grenada	93.48	81.25, 106.04	114.36	97.09, 132.66	132.91	118.34, 147.89	136.72	118.52, 155.92	65.58	54.99, 76.45	94.67	78.29, 112.09
Guatemala	101.36	85.71, 117.85	85.23	66.30,105.95	113.77	95.62, 133.01	98.12	76.55, 122.80	90.59	77.12, 104.72	74.75	58.01, 92.23
Guyana	215.18	180.32, 252.79	192.88	147.90, 259.09	250.84	210.23, 296.28	228.89	175.84, 312.72	180.59	151.47, 210.55	157.59	120.67, 206.09
Honduras	115.72	77.82, 165.23	129.16	89.30, 177.65	114.00	74.98, 166.38	142.54	99.03, 194.95	116.22	79.43, 163.25	118.26	81.30, 163.68
Haiti	199.63	132.77, 282.24	185.42	114.89, 276.57	203.31	141.04, , 285.85	190.32	124.63, 278.85	196.45	125.88, 279.25	180.35	106.35, 273.21
Jamaica	52.32	44.47. 59.39	49.67	38.41, 62.16	60.62	52.67, 67.93	54.31	42.35, 67.47	44.93	37.18, 51.79	45.39	34.76, 57.26
Saint Lucia	80.01	68.29, 91.90	60.14	47.88, 73.18	100.49	86.95, 114.29	76.68	62.02, 92.31	61.39	51.37, 71.53	44.65	34.63, 55.27
Mexico	83.05	76.37, 95.69	92.37	74.93, 1, 8.58	98.27	91.29, 113.30	116.67	94.27, 149.92	69.89	63.46, 80.06	71.89	58.60, 91.92
Nicaragua	132.47	113.50, 178.28	124.42	96.81, 181.43	147.64	128.30, 174.80	152.47	118.36, 220.97	119.63	101.24, 184.81	103.84	81.23, 150.82
Panama	69.07	59.11, 80.33	53.53	39.42, 73.28	83.25	73.06, 98.33	67.42	49.98, , 92.51	55.64	46.00, 62.91	40.88	29.87, 55.59
Peru	54.61	42.88, 67.54	39.95	25.78, 57.18	60.28	47.72, 74.14	46.57	30.41, 66.57	49.71	38.76, 61.74	33.73	21.51, 48.24
Paraguay	84.94	68.54, 123.19	76.64	53.88, 107.75	101.12	82.36, 148.19	97.12	68.81, 136.48	70.08	55.90, 99.30	56.96	39.67, 79.96
El Salvador	116.29	97.04, 170.77	82.52	59.20, 134.39	138.70	117.75, 195.65	105.20	75.83, 166.32	99.19	81.34, 151.51	66.26	47.35, 110.85
Suriname	124.31	103.31, 146.19	123.23	89.62, 161.75	156.21	132.00, 181.99	164.62	121.17, 215.24	96.97	78.81, 115.50	90.20	64.56, 118.96

Trinidad and Tobago	179.01	161.87, 194.46	81.57	60.84, 105.92	225.00	206.38, 243.12	109.33	81.79, 141.98	141.67	125.84, 155.00	58.89	43.93, 76.12
Uruguay	93.15	83.72, 107.44	59.79	51.92, 78.87	130.43	119.76, 146.31	88.36	78.78, 106.04	65.03	56.76, 78.18	39.45	32.91, 60.52
United States of America	128.88	118.93, 147.75	73.47	66.14, 89.97	167.95	159.05, 187.77	97.80	90.21, 114.12	97.67	87.46, 115.20	52.53	45.63, 69.08
Saint Vincent and the Grenadines	134.40	116.92, 150.56	93.70	78.10, 109.99	147.55	130.17, 164.14	99.38	84.06, 115.44	122.89	105.52, 138.54	87.36	71.68, 103.64
Venezuela	128.76	115.06, 142.90	99.29	74.16, 128.90	164.95	150.38, 181.78	130.85	98.62, 169.09	98.07	85.32, 109.53	74.07	54.93, 96.34

Table 2S. Joinpoint analysis for ischemic heart diseases mortality trends in men in the region of the Americas countries from 2000 to 2019

Country	Total study period	Period 1		Period 2		Period 3	
	AAPC (95% CI)	Years	APC (95% CI)	Years	APC (95% CI)	Years	APC (95% CI)
Antigua and Barbuda	-1.0 (-2.0, 0.1)	2000-2006	4.2* (1.0, 7.5)	2006-2019	-3.3* (-4.2, -2.3)		
Argentina	-2.5* (-2.8, -2.2)						
Bahamas	-0.8* (-1.5, -0.2)	2000-2014	-1.7* (-2.1, -1.2)	2014-2019	1.6 (-0.7, 4.0)		
Barbados	-2.1 (-4.5, 0.4)	2000-2009	-2.3* (-3.7, -0.8)	2009-2012	-9.8 (-23.5, 6.3)	2012-2019	1.6 (-0.6, 3.9)
Belize	-3.7* (-5.0, -2.3)	2000-2002	-15.6* (-25.3, -4.6)	2002-2012	-4.3* (-5.4, -3.3)	2012-2019	1.0 (-0.7, 2.6)
Bolivia	-0.6* (-0.9, -0.2)	2000-2005	-2.6* (-3.1, -2.1)	2005-2008	-0.9 (-3.1, 1.4)	2008-2019	0.5* (0.3, 0.6)
Brazil	-2.2* (-2.3, -2.1)						
Canada	-4.0* (-4.2, -3.8)	2000-2011	-5.2* (-5.4, -4.9)	2011-2019	-2.4* (-2.7, -2.0)		
Chile	-3.2* (-3.3, -3.0)						
Colombia	-0.9 (-2.3, 0.5)	2000-2006	-0.4 (-2.0, 1.1)	2006-2009	-3.8 (-12.3, 5.5)	2009-2019	-0.3 (-1.0, 0.4)
Costa Rica	-3.9* (-4.9, -2.8)	2000-2007	-5.6* (-7.1, -4.0)	2007-2015	-0.6 (-2.2, 1.0)	2015-2019	-7.1* (-10.7, -3.4)
Cuba	-1.4* (-2.0, -0.7)	2000-2013	-2.3* (-2.9, -1.7)	2013-2019	0.6 (-1.3, 2.5)		
Dominican Republic	1.9* (0.5, 3.3)	2000-2002	-2.7 (-12.7, 8.6)	2002-2016	4.1* (3.5, 4.7)	2016-2019	-5.2 (-10.2, 0.1)
Ecuador	-0.6* (-1.2, -0.1)	2000-2005	2.6* (1.2, 4.1)	2005-2014	-2.3* (-3.0, -1.7)	2014-2019	-0.7 (-2.0, 0.7)
El Salvador	-1.0* (-1.8, -0.2)						
Grenada	0.2 (-1.4, 1.9)	2000-2005	6.5* (0.4, 12.9)	2005-2019	-1.9* (-3.1, -0.7)		
Guatemala	-0.8* (-1.6, -0.0)	2000-2002	-4.4 (-10.8, 2.4)	2002-2015	0.4* (0.0, 0.8)	2015-2019	-3.0* (-5.1, -0.9)
Guyana	-0.4 (-0.8, 0.0)	2000-2005	1.5 (-0.1, 3.1)	2005-2019	-1.1* (-1.4, -0.7)		
Haiti	-0.2 (-0.6, 0.1)	2000-2003	-1.5 (-3.5, 0.5)	2003-2019	-0.0 (-0.2, 0.1)		
Honduras	1.0 (-0.1, 2.1)	2000-2010	-0.3 (-0.8, 0.3)	2010-2013	15.7* (7.8, 24.2)	2013-2019	-3.6* (-4.7, -2.4)
Jamaica	-0.9 (-4.6, 3.0)	2000-2005	-6.0* (-11.0, -0.7)	2005-2008	10.1 (-13.8, 40.7)	2008-2019	-1.3 (-2.9, 0.3)
Mexico	0.9* (0.5, 1.3)	2000-2007	-0.6 (-1.5, 0.4)	2007-2019	1.8* (1.3, 2.2)		
Nicaragua	0.5 (-1.6, 2.7)	2000-2003	5.2 (-1.5, 12.4)	2003-2006	-3.8 (-15.7, 9.7)	2006-2019	0.5 (-0.2, 1.2)

Panama	-1.3* (-2.0, -0.6)	2000-2010	0.6 (-0.5, 1.6)	2010-2019	-3.4* (-4.5, -2.2)		
Paraguay	0.3 (-0.6, 1.2)	2000-2010	1.9* (0.6, 3.2)	2010-2019	-1.4 (-2.9, 0.1)		
Peru	-1.5* (-2.5, -0.5)	2000-2007	-3.4* (-4.2, -2.5)	2007-2010	4.9 (-1.9, 12.0)	2010-2019	-2.1* (-2.7, -1.5)
Saint Lucia	-1.3* (-1.8, -0.7)	2000-2003	0.1 (-2.6, 2.8)	2003-2012	-4.1* (-4.6, -3.5)	2012-2019	1.9* (1.2, 2.7)
Saint Vincent	-2.4* (-4.4, -0.3)	2000-2004	-3.9 (-7.9, 0.2)	2004-2007	8.9 (-4.6, 24.4)	2007-2019	-4.5* (-5.2, -3.7)
Suriname	0.2 (-0.4, 0.8)	2000-2002	1.1 (-3.8, 6.2)	2002-2012	-2.4* (-2.8, -1.9)	2012-2019	3.7* (3.1, 4.4)
Trinidad and Tobago	-4.0* (-4.3, -3.7)						
United States of America	-2.8* (-3.0, -2.7)	2000-2011	-4.2* (-4.4, -4.1)	2011-2019	-0.9* (-1.2, -0.6)		
Uruguay	-2.2* (-2.5, -2.0)						
Venezuela	-1.0* (-1.5, -0.4)	2000-2014	-0.3 (-0.7, 0.1)	2014-2019	-2.8* (-4.6, -1.0)		

AAPC, average annual percent change; APC, annual percent change; CI, confidence interval. *P < 0.05 for change in trend.

Table 3S. **Joinpoint analysis for ischemic heart diseases mortality trends in women in the region of the Americas countries from 2000 to 2019**

Country	Total study period	Period 1		Period 2		Period 3	
	AAPC (95% CI)	Years	APC (95% CI)	Years	APC (95% CI)	Years	APC (95% CI)
Antigua and Barbuda	-0.1 (-2.0, 1.8)	2000-2009	4.2* (2.3, 6.0)	2009-2014	-8.6* (-14.0, -2.8)	2014-2019	1.1 (-3.1, 5.6)
Argentina	-2.9* (-4.0, -1.8)	2000-2005	-3.9* (-6.3, -1.4)	2005-2010	-0.4 (-3.9, 3.2)	2010-2019	-3.7* (-4.7, -2.7)
Bahamas	-1.3* (-1.7, -0.9)	2000-2007	-2.8* (-3.7, -1.9)	2007-2019	-0.4 (-0.8, 0.0)		
Barbados	-1.8* (-2.7, -0.9)	2000-2006	-0.9 (-2.5, 0.6)	2006-2011	-5.6* (-8.3, -2.8)	2011-2019	0.0 (-0.9, 1.0)
Belize	-3.2* (-3.8, -2.6)	2000-2008	-4.9* (-5.6, -4.2)	2008-2016	-3.1* (-4.0, -2.3)	2016-2019	1.1 (-2.2, 4.5)
Bolivia	-0.4* (-0.5, -0.2)	2000-2004	-2.5* (-2.9, -2.0)	2004-2016	0.4* (0.3, 0.5)	2016-2019	-0.5 (-1.2, 0.2)
Brazil	-3.0* (-3.3, -2.8)	2000-2007	-3.5* (-4.1, -3.0)	2007-2019	-2.8* (-3.0, -2.5)		
Canada	-4.2* (-4.4, -4.1)	2000-2012	-5.3* (-5.4, -5.1)	2012-2019	-2.4* (-2.8, -2.0)		
Chile	-4.0* (-4.4, -3.7)						
Colombia	-1.5* (-1.7, -1.3)						
Costa Rica	-4.6* (-6.1, -3.0)	2000-2009	-5.3* (-6.8, -3.9)	2009-2014	0.5 (-4.6, 5.9)	2014-2019	-8.0* (-11.3, -4.6)
Cuba	-1.9* (-2.5, -1.2)	2000-2013	-2.6* (-3.2, -2.0)	2013-2019	-0.3 (-2.1, 1.6)		
Dominican Republic	2.7* (2.1, 3.3)	2000-2005	5.2* (3.8, 6.6)	2005-2016	3.0* (2.5, 3.5)	2016-2019	-2.7 (-5.6, 0.3)
Ecuador	-1.1* (-1.7, -0.4)	2000-2006	0.3 (-0.8, 1.4)	2006-2011	-2.8* (-4.8, -0.7)	2011-2019	-1.0* (-1.7, -0.3)
El Salvador	-1.9* (-2.6, -1.2)						
Grenada	2.4* (1.3, 3.4)	2000-2006	5.2* (2.1, 8.4)	2006-2019	1.1* (0.2, 2.0)		
Guatemala	-1.0* (-1.7, -0.4)	2000-2002	-4.7 (-10.0, 1.0)	2002-2015	-0.2 (-0.5, 0.2)	2015-2019	-1.9* (-3.6, -0.1)
Guyana	-0.7* (-1.1, -0.4)	2000-2004	2.0* (0.7, 3.4)	2004-2014	-2.4* (-2.8, -2.1)	2014-2019	0.5 (-0.4, 1.4)
Haiti	-0.5* (-0.6, -0.3)						
Honduras	0.1 (-0.2, 0.5)	2000-2009	-0.1 (-0.3, 0.1)	2009-2012	3.3* (1.1, 5.6)	2012-2019	-0.9* (-1.2, -0.6)
Jamaica	0.1 (-1.0, 1.2)	2000-2005	-4.6* (-7.0, -2.2)	2005-2010	0.4 (-3.1, 4.0)	2010-2019	2.6* (1.6, 3.7)
Mexico	0.2 (-0.2, 0.6)	2000-2007	-1.5* (-2.5, -0.6)AA	2007-2019	1.2* (0.8, 1.6)		
Nicaragua	-1.0 (-3.1, 1.2)	2000-2011	-1.1* (-2.0, -0.2)	2011-2014	2.9 (-10.2, 18.0)	2014-2019	-2.9 (-5.8, 0.1)

Panama	-1.7* (-2.5, -0.8)	2000-2010	0.0 (-1.2, 1.3)	2010-2019	-3.6* (-5.0, -2.2)		
Paraguay	-1.1 (-2.6, 0.5)	2000-2003	-3.6 (-10.6, 4.0)	2003-2009	2.4 (-1.0, 6.0)	2009-2019	-2.4* (-3.5, -1.2)
Peru	-2.2* (-3.3, -1.1)	2000-2007	-4.6* (-5.6, -3.7)	2007-2010	3.2 (-4.3, 11.2)	2010-2019	-2.0* (-2.7, -1.4)
Saint Lucia	-1.6* (-2.1, -1.1)	2000-2005	-2.5* (-3.6, -1.3)	2005-2010	-6.4* (-7.9, -4.8)	2010-2019	1.7* (1.2, 2.2)
Saint Vincent	-1.9 (-4.3, 0.6)	2000-2005	-2.3 (-5.7, 1.3)	2005-2008	14.3 (-2.5, 34.0)	2008-2019	-5.7* (-6.7, -4.7)
Suriname	-0.5* (-0.9, -0.1)	2000-2008	-3.0* (-3.4, -2.5)	2008-2014	-1.4* (-2.3, -0.4)	2014-2019	4.7* (3.7, 5.7)
Trinidad and Tobago	-4.6* (-4.8, -4.4)						
United States of America	-3.3* (-3.7, -2.8)	2000-2011	-4.8* (-5.0, -4.5)	2011-2017	-1.9* (-2.8, -1.0)	2017-2019	1.2 (-2.8, 5.4)
Uruguay	-2.9* (-3.4, -2.5)						
Venezuela	-1.3* (-1.5, -1.0)						

AAPC, average annual percent change; APC, annual percent change; CI, confidence interval. *P < 0.05 for change in trend.
